# Effects of Free Linoleic Acid and Oleic Acid in Sesame Meal Extract as Pancreatic Lipase Inhibitors on Postprandial Triglyceridemia: A Randomized, Double-Blind, Placebo-Controlled, Crossover Study in Healthy Volunteers

**DOI:** 10.3390/nu15071748

**Published:** 2023-04-03

**Authors:** Xuan Li, Hiroaki Yamada, Sayo Morita, Yusuke Yamashita, Youngil Kim, Takashi Kometani, Nikesh Narang, Toma Furuta, Mujo Kim

**Affiliations:** 1 Pharma Foods International Co., Ltd., Kyoto 615-8245, Japan; 2 Mitsui DM Sugar Co., Ltd., Tokyo 100-0011, Japan

**Keywords:** pancreatic lipase inhibitor, clinical trial, linoleic acid, oleic acid, obesity, dietary fat absorption, postprandial triglyceridemia, postprandial lipemia, atherogenic lipoproteins, functional food

## Abstract

A great number of chemically diverse pancreatic lipase (PL) inhibitors have been identified to tackle obesity; however, very few of them have entered clinical studies. The ethanolic extract of sesame meal is a potent PL inhibitor, and its activity hinges exclusively on two free fatty acids: linoleic acid and oleic acid, which were proven to reduce postprandial triglyceride excursion in rats. Herein, to investigate the clinical efficacy of the sesame meal extract, in a crossover trial, 30 healthy volunteers were randomized to receive the sesame meal extract containing experimental food or placebo along with a high-fat meal. Treatment with the sesame meal extract significantly lowered the incremental postprandial serum triglyceride concentration and reduced the incremental area under the curve (iAUC) by 16.8% (*p*-value = 0.03) compared to placebo. Significant decreases in postprandial remnant-like lipoprotein particle cholesterol and low-density lipoprotein particles were also observed, whereas high-density lipoprotein cholesterol was increased. These results suggest that treatment with the sesame meal extract significantly reduced the postprandial excursion of triglycerides and improved the lipidemic profile after high dietary fat intake in healthy individuals, indicating the substantial potential of free linoleic acid and oleic acid and natural products rich in these compounds for the management of obesity and related conditions.

## 1. Introduction

The past decades have seen an ever-worsening public health crisis worldwide—the obesity epidemic. Obesity increases the risk of a range of chronic conditions that often lead to severely impaired quality of life and premature mortality. Epidemiological studies have revealed a direct relationship between the incidence of overweight/obesity and dietary fat intake, which, in most populations, has exceeded the recommended <30% of total energy intake [1]. Excessive fat absorption also fuels the formation and accumulation of atherogenic lipoproteins [2]. Dietary fat is ingested primarily in the form of triglycerides (TGs). The digestion of TGs relies to a great extent on pancreatic lipase (PL), which is recognized as the single most important determinant of dietary fat absorption [3]. Indeed, PL inhibition is one of the most widely investigated strategies to tackle obesity, resulting in the reports of a great number of chemically diverse PL inhibitors derived from a wide variety of sources, in particular, natural products [4]. Targeting PL is generally considered safe, as PL inhibitors do not alter any central mechanisms or enter the systemic circulation [5]. Nonetheless, to date, orlistat remains the only PL inhibitor approved for clinical use to aid weight loss with known gastrointestinal adverse effects. Moreover, since orlistat is indicated for patients with a body mass index (BMI) of 30 kg/m^2^ or greater and for those who have a BMI over 27 kg/m^2^ and obesity-associated comorbidities [6], it can hardly be used by healthy, normal-weight individuals for weight management purposes. Aside from orlistat, despite the tremendous number of compounds that demonstrate PL inhibitory activities in preclinical screenings, very few of them have entered clinical studies. This is likely due to the low abundance of these active compounds in natural products, complex extraction procedures, and low recovery rate, which collectively remain a critical hurdle to the commercialization of the PL inhibitors from natural sources [5].

Sesame (*Sesamum indicum* L.) seeds and related food products are commonly consumed by East Asian populations. Sesame seed is well recognized as a valuable reservoir of biologically active and health-promoting phytochemicals [7]. The byproduct of sesame oil pressing, known as sesame meal, remains a rich, cost-effective and low-calorie source of bioactive compounds. We reported earlier that the ethanolic extract of sesame meal exhibited strong inhibitory effects on porcine PL in vitro; a bioactivity-guided fractionation of the extract further revealed that its PL inhibitory activity hinged exclusively on two non-esterified free fatty acids (FFAs): linoleic acid (LA, IC_50_ = 23.1 µg/mL or 82.4 µM) and oleic acid (OA, IC_50_ = 11.7 µg/mL or 41.4 µM). A mixture of these FFAs was then proven to be effective in lowering postprandial TG excursion following an oral fat load in rats [8]. In the present study, we aimed to investigate the clinical efficacy of the sesame meal extract as a carrier of free LA and OA in normalizing postprandial triglyceridemia and other lipidemic parameters in healthy individuals and to discuss the potential use of free LA and OA and the natural products rich in these FFAs in the development of novel functional food for obesity management for the general population.

## 2. Materials and Methods

### 2.1. Sesame Meal Extract

The sesame meal extract used for the trial was prepared following the method reported previously [8] with modifications. Specifically, 150 g of sesame meal (Mitsui DM Sugar Co., Ltd., Tokyo, Japan) was extracted with 20 volumes (*v*/*w*) of 50% ethanol (Wako, Osaka, Japan) at 30 °C for 3 h; the resulting soluble part was clarified by filtration (Qualitative filter paper No. 2, Whatman, Maidstone, UK) and lyophilized after the ethanol was removed by rotary evaporation. This process yielded 20.5 g (13.7%) of sesame meal extract.

### 2.2. Quantification of Free LA and OA in the Sesame Meal Extract

The sesame meal extract was dissolved in 50% ethanol to 10 mg/mL, clarified with 0.45 µm syringe filters, and 10 μL of the sample was injected into a Shimadzu HPLC system (Shimadzu, Kyoto, Japan) with a COSMOSIL Cholester packed column (4.6 mm I.D. × 250 mm, Nacalai, Kyoto, Japan), eluted at 1 mL/min, 40 °C. A binary gradient elution was employed with 0.1% (*v*/*v*) acetic acid (Nacalai, Kyoto, Japan) aqueous solution and 95% acetonitrile (Nacalai, Kyoto, Japan) with 0.1% (*v*/*v*) acetic acid as mobile phase A and B, respectively. The gradient of mobile phase B was programed as: 0–5 min, 5% to 20%; 5–40 min, 20% to 60%; 40–50 min, 100%; 50–65 min, 5%. UV detection was carried out at 195 nm. The contents of free LA and OA in the sesame meal extract were quantified by using the standard curves generated with reference standards (Wako, Osaka, Japan).

### 2.3. Lipase Inhibition Assay

The lipase inhibition assay was performed in accordance with the method previously reported [8]. First, 0.06 mg/mL enzyme solution was prepared by reconstituting the lipase from porcine pancreas (Type II, Sigma-Aldrich, St. Louis, United States) in 0.1 M citrate buffer (pH 6.0). The activities of the sesame meal extract and the mixtures of free LA and OA against pancreatic lipase were measured by using a Lipase Kit S (SB Bioscience, Osaka, Japan) following the manufacturer’s instructions. All samples were dissolved in dimethyl sulfoxide (DMSO) and DMSO was used as negative control and blank. In addition, 0.29 ng/mL orlistat was used as positive control. Then, a 100 μL sample was incubated with 9 μL reconstituted lipase solution in the presence of 4 μL esterase inhibitor (phenylmethylsulfonyl fluoride) and 237 μL color-developing agent (5,5′-dithiobis-(2-nitrobenzoic acid)) at 30 °C for 5 min. Afterwards, 25 μL substrate solution (2,3-dimercapto-1-propanol-tributyrate) was then added to the mixture and incubated in the dark for 30 min before the reaction was quenched with 700 μL stop solution. Absorbance measurement was taken at 412 nm. To determine the background absorbance of the reaction components, the sample, the lipase solution, the esterase inhibitor and the color-developing agent were mixed and incubated in the dark for 35 min, which was followed by the addition of the stop solution and the substrate, and then, the absorbance at 412 nm was measured. Pancreatic lipase activity was calculated according to the following equation:

Pancreatic lipase activity (%) = (absorbance412 of sample−background

absorbance412 of sample)/(absorbance412 of blank−background

absorbance412 of blank) × 100.

### 2.4. Experimental Food and the Standardized High-Fat Meal

The experimental food was composed of 90 mg sesame meal extract and 810 mg dextrin (maltodextrin, dextrose equivalent 15~18, Sanwa Starch Co., Ltd., Nara, Japan) per serving. Here, 900 mg dextrin was used as placebo per serving. The nutritional composition of the experimental food and the placebo is provided in Table 1. Food coloring was used to make the experimental food and the placebo indistinguishable in appearance for the investigators and participants. The standardized high-fat meal was made of commercial products, as detailed in Table 2. Each participant was also provided with an identical meal as dinner on the day prior to each intervention. The meal was a salmon deluxe bento purchased from a local restaurant with a macronutrient breakdown of 22.0 g fat, 28.7 g protein, and 103.2 g carbohydrate and a total calorie content of 747 kcal.

The compositional analysis (except the contents of free linoleic acid and oleic acid) was conducted by the Japan Food Research Laboratories.

### 2.5. Ethics Statement

The protocol was approved by the Ethics Committees of Nihonbashi Egawa Clinic (Tokyo, Japan; Ethical approval code: 16000016), and the trial was conducted in accordance with the Declaration of Helsinki and ICH Good Clinical Practice. Participants were provided with both oral and written information about the study. Written informed consent was obtained from all participants prior to the study. The study was registered at UMIN Clinical Trials Registry (UMIN-CTR) (UMIN000037160).

### 2.6. Participants

Healthy Japanese volunteers aged 20–65 years with fasting triglyceride levels of <150 mg/dL and BMI of 18.5–27 kg/m^2^ based on the results of the latest annual physical examinations and the ability to give informed consent were considered eligible for the study. Subjects who met any of the following criteria were excluded: (1) history of malignancy, heart failure and myocardial infarction; (2) history of treatment or currently receiving treatment for the following chronic diseases: atrial fibrillation, arrhythmia, liver disorder, kidney disorder, cerebrovascular disorder, rheumatism, diabetes, dyslipidemia, hypertension, etc.; (3) frequent use of oral medications, nutritional supplements or functional foods (including traditional Chinese medicine) that could affect lipid metabolism; (4) history of allergic reactions to medical products or foods/food ingredients related to the foods used in this study; (5) females who were pregnant or breastfeeding or who were planning a pregnancy; (6) other conditions considered unsuitable for the study by the physician. The use of medicines for minor illnesses (e.g., colds, headaches, etc.) were allowed and documented during the study. A total of 31 subjects (male 18, female 13) were eligible and enrolled in the study based on the inclusion and exclusion criteria.

### 2.7. Study Design and Procedures

The study was conducted at Pharma Foods International Co., Ltd. (Kyoto, Japan). It was a randomized, double-blind, placebo-controlled, crossover trial. In this crossover trial, the participants were randomly allocated to the experimental food–placebo sequence or the placebo–experimental food sequence based on the random numbers (generated in Excel) assigned to each of them. The random allocation was performed by an independent third party not involved in the study, and the allocation sequence was concealed before and until assignment to prevent selection bias. The allocation was blinded to the participants, investigators, caregivers and outcome assessors. The two interventions of each sequence were separated by a washout period of one week. On the day prior to each intervention, each participant was given an identical meal as dinner and asked to refrain from consuming any food or beverage except water from 21:00 until 9:00 the following morning. On the day of intervention, which started shortly after 9:00, the participants were instructed to self-administer the experimental food or placebo with drinking water and then to consume the standardized high-fat meal within 15 min. Blood samples were collected before intake of the experimental food/placebo (0 h) and at 2 h, 3 h, 4 h, and 6 h after consumption of the high-fat meal. Throughout the study, the participants were instructed to maintain their habitual diet and physical activity patterns. The participants were also required not to start to use any functional foods or nutritional supplements (i.e., expect those routinely used) other than the experimental food/placebo during the study. On the day prior to each intervention, the participants were asked to refrain from alcohol intake and strenuous physical activity. Moreover, from the day prior to the first intervention until completion of the second intervention, any use of medicines or functional foods or nutritional supplements was documented on a daily basis. Adverse events were monitored via participant interview by the physician at the time of blood specimen collection and self-reports during the study.

The primary endpoints of this study were the differences in postprandial serum TG levels at sequential sampling time points and the incremental area under the curve (iAUC) of postprandial serum TGs between the two intervention arms. Secondary outcome measures include the differences in postprandial serum remnant-like lipoprotein particle (RLP) cholesterol levels at sequential sampling time points and the RLP cholesterol iAUC between the intervention arms, and the differences in other lipid metabolism-related parameters, including FFAs, total cholesterol, phospholipids, beta lipoproteins (βLPs), high-density lipoprotein (HDL) cholesterol, low-density lipoprotein (LDL) cholesterol, and blood glucose. The safety endpoint of this trial was the occurrence of adverse events.

### 2.8. Blood Specimen Collection

Venous blood samples were collected from antecubital fossa into 6 mL vacuum blood collection tubes (Terumo, Tokyo, Japan). The serum tube was allowed to clot for 30 min at room temperature before centrifugation (1200× *g*, 15 min). Serum aliquots were stored at −80 °C until analysis.

### 2.9. Biochemical Analysis

The concentrations of TGs in serum were determined using an enzymatic colorimetric assay (Pure Auto TG-N; Sekisui Medical, Tokyo, Japan). The measurement of phospholipids in serum was based on the choline oxidase DAOS (N-Ethyl-N-(2-hydroxy-3-sulfopropyl)-3,5 dimethoxyaniline sodium salt) method (L-Type Phospholipids; Wako, Osaka, Japan). Quantitative analysis of total cholesterol was performed using an enzymatic colorimetric assay (Cholestest CHO; Sekisui Medical, Tokyo, Japan). The serum concentrations of βLPs were measured using a turbidimetric assay (Clinimate β-L; Sekisui Medical, Tokyo, Japan). A direct method was used to determine the serum concentrations of HDL cholesterol (Cholestest N HDL; Sekisui Medical, Tokyo, Japan). An enzymatic colorimetric assay (Cholestest LDL; Sekisui Medical, Tokyo, Japan) was employed to quantify LDL cholesterol in serum. The above assays were performed on a Labospect 008 automatic biochemical analyzer (Hitachi, Ibaraki, Japan). Non-esterified FFAs in serum were quantitatively determined using an enzymatic assay based on the ACS-ACOD (acyl CoA synthetase–acyl CoA oxidase) method (NEFA-HR (2); Wako, Osaka, Japan). RLP cholesterol levels were measured using a homogeneous assay (MetaboLead RemL-C; Kyowa Medex, Tokyo, Japan). Measurements of FFAs and RLP cholesterol were conducted on a JCA-BM8040 automatic analyzer (JEOL, Tokyo, Japan). Blood glucose was measured based on the kinetic-spectrophotometric method (CicaLiquid Glu J; Kanto Chemical, Tokyo, Japan) by using a JCA-BM9130 automatic analyzer (JEOL, Tokyo, Japan).

### 2.10. Statistical Analysis

Based on the data of a preliminary experiment (data not published), this trial was >80% powered to detect a 0.8 standard deviation difference in the TG iAUC endpoint between the intervention arms, given a two-sided, Type I error of 5%. Hence, it was determined that approximately 30 subjects were required, assuming a dropout rate of 12%. Postprandial levels of TGs and RLP cholesterol at sequential time points were expressed as incremental values, which were calculated by subtracting the baseline value from that of each time point. Differences between the intervention arms in these variables at sequential time points were analyzed by using two-tailed, paired *t*-tests. iAUCs were calculated by applying the trapezoidal rule after subtracting the baseline value from the postprandial values. The effects of the experimental food vs. placebo on the iAUCs of TGs and RLP cholesterol were evaluated by using mixed-effects models with intervention, gender, age, BMI and sequence as fixed variables and subject as a random effect. Logarithmic transformations were performed for the iAUCs of TG and RLP cholesterol to satisfy the assumption of normality. Residuals from the model were visually checked for approximately normal distributions and homoscedasticity over predictions. Carryover effects were insignificant (*t*-test, *p*-value = 0.66 for TG iAUC) and thus not included in the final models. Postprandial levels of other secondary variables at sequential time points were analyzed by using two-tailed, paired *t*-tests. Statistical analysis was performed on the per protocol set with R version 4.2.1. Statistical significance was established at *p*-value ≤ 0.05. Values are mean ± standard error (SE) except the baseline characteristics of participants, which are expressed as mean ± standard deviation (SD). A final statistical analysis plan was submitted, and the conditions for excluding randomized and exposed participants were specified prior to unblinding.

## 3. Results

### 3.1. In Vitro Pancreatic Lipase Inhibitory Activity of the Sesame Meal Extract

**Figure 1:**
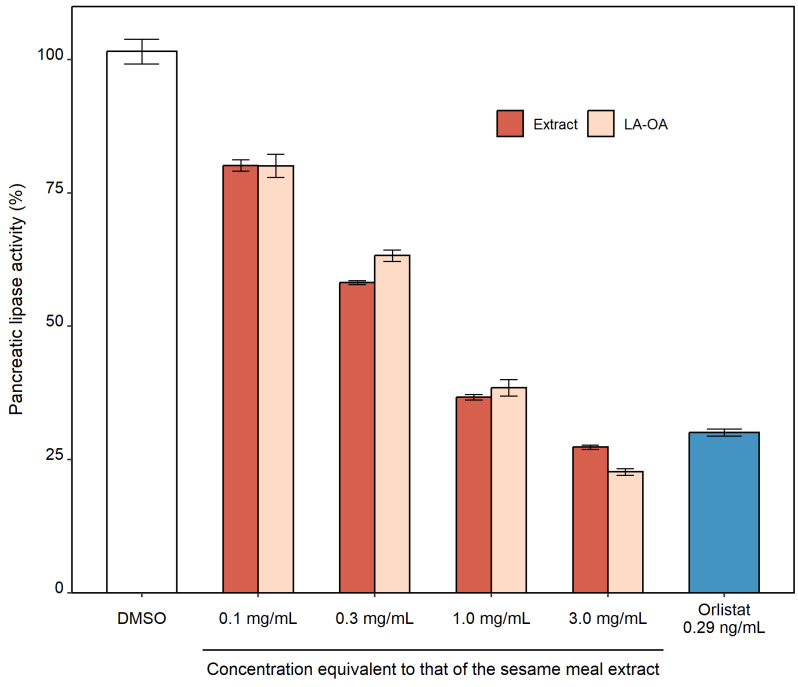
In vitro pancreatic lipase inhibitory activities of the sesame meal extract and the mixtures of free linoleic acid (LA) and oleic acid (OA). Values are mean ± standard error (SE) (*n* = 3). The mixture of LA and OA (LA-OA) were composed of 2.3 and 2.1 μg/mL, 6.9 and 6.3 μg/mL, 23 and 21 μg/mL, and 69 and 63 μg/mL of free LA and OA, respectively, which were equal to the amount contained in 0.1, 0.3, 1.0 and 3.0 mg/mL of the sesame meal extract (Extract). Dimethyl sulfoxide (DMSO) and 0.29 ng/mL orlistat was used as negative and positive control, respectively.

Quantitative analysis revealed that the sesame meal extract used for the clinical study contained 2.3% of LA and 2.1% of OA in their non-esterified forms. The PL-inhibitory activities of the sesame meal extract and the mixtures of free LA and OA (LA-OA) at various concentrations were determined in vitro, and the results are presented in Figure 1. DMSO, the solvent used to dissolve the samples, did not affect PL activity. Orlistat, the positive control, exhibited significant PL inhibitory activity at 0.29 ng/mL, suggesting that the assay was performed properly. The LA-OA mixtures were composed of 2.3 and 2.1 μg/mL, 6.9 and 6.3 μg/mL, 23 and 21 μg/mL, and 69 and 63 μg/mL of free LA and OA, respectively, which were equal to the amount contained in 0.1, 0.3, 1.0 and 3.0 mg/mL of the sesame meal extract. Both the LA-OA mixtures and the sesame meal extract exhibited increasing inhibitory activities against porcine PL as their concentrations were increased. Importantly, the PL-inhibitory activities of the LA-OA mixtures and the sesame meal extract did not differ across the range of concentrations investigated (two-way ANOVA, *p*-value = 0.5192), suggesting that the activity of the sesame meal extract can be solely attributed to the presence of free LA and OA.

### 3.2. Subject Demographics

**Figure 2:**
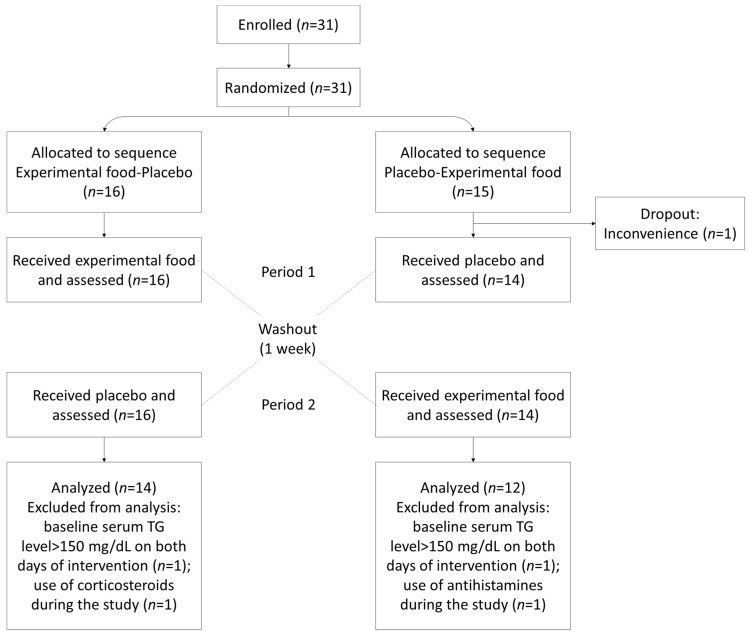
Flow diagram of the trial. The schema graphically outlines the design and conduct of the two-arm, two-period crossover trial. TG, triglyceride.

A total of 31 eligible healthy volunteers consented to participate in the study and were randomized. One withdrew before the start of intervention; therefore, 30 participants were assigned to the experimental food–placebo sequence (*n* = 16: male 12; female 4) or the placebo–experimental food sequence (*n* = 14: male 6; female 8) (Table 3). The postprandial TG and other lipid parameter endpoints were excluded for two participants due to the use of oral corticosteroids (from the experimental food–placebo sequence) or antihistamines (from the placebo–experimental food sequence) and for two due to fasting TG level >150 mg/dL (one from each sequence) on both days of intervention and assessment. Accordingly, the per protocol set consisted of 26 subjects (the experimental food–placebo sequence: *n* = 14, male 10, female 4; the placebo–experimental food sequence: *n* = 12, male 4, female 8) (Figure 2).

### 3.3. Postprandial TG Responses

**Figure 3:**
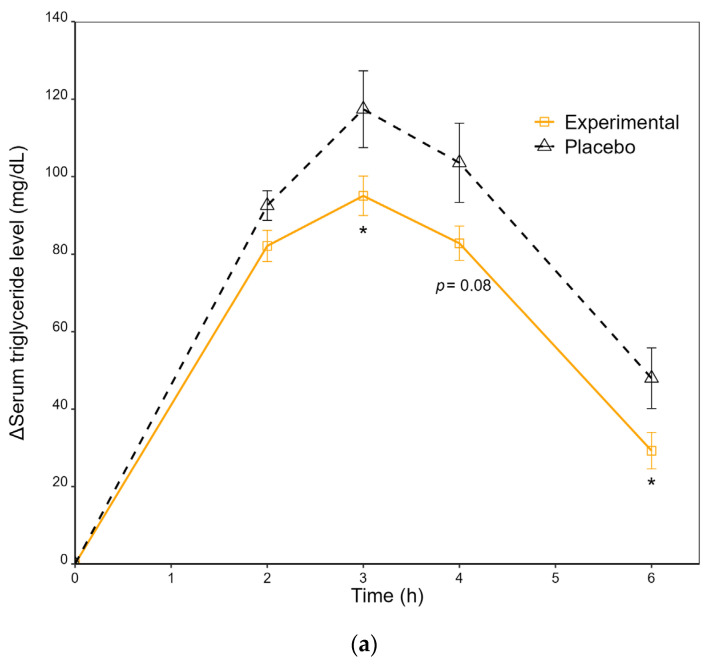

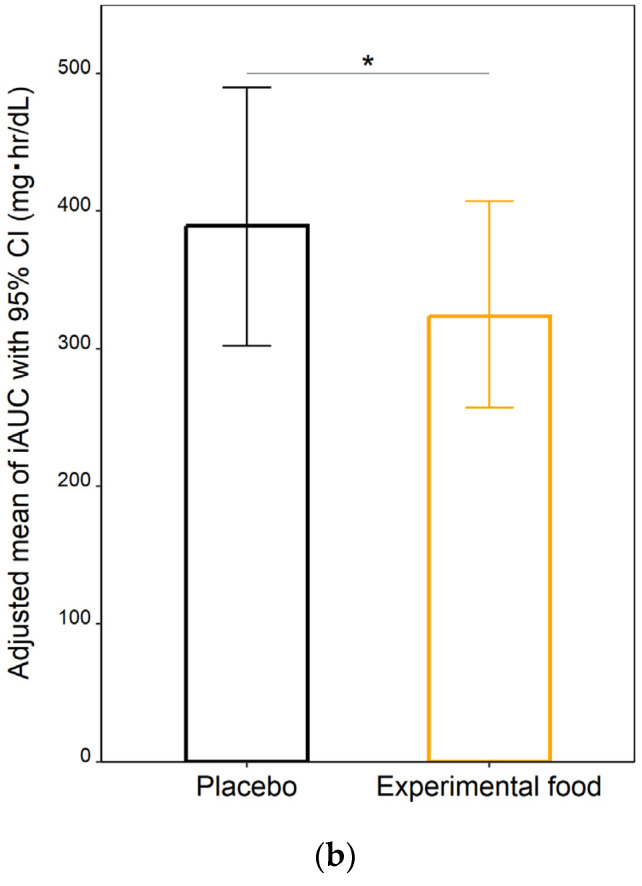
Postprandial triglyceride (TG) responses to ingestion of the experimental food or placebo and the standardized high-fat diet. (**a**) Time course of the incremental postprandial serum TG concentrations. Values are mean ± standard error (SE). Statistical analysis by two-tailed, paired *t*-tests. * *p*-value < 0.05. (**b**) Incremental area under the curve (iAUC) of postprandial serum TGs. Values are covariate-adjusted mean ± 95% confidence interval (CI) and were compared by using a mixed-effects model with intervention, gender, age, body mass index (BMI) and sequence as fixed variables and subject as a random effect. * *p*-value < 0.05.

The postprandial TG responses, which were measured as the incremental serum levels of TGs relative to the baseline/fasting values (denoted by Δ) at sequential time points and the iAUCs of TGs, are illustrated in Figure 3. The baseline/fasting serum levels of TGs were 78.0 ± 5.9 mg/dL and 88.5 ± 9.0 mg/dL for the experimental food and placebo arm, respectively. For both intervention arms, the incremental levels of TGs increased drastically in response to a high dietary fat intake and peaked at 3 h. The experimental food arm exhibited a slower increase in serum TGs, resulting in a significantly reduced peak value of the incremental serum TG concentration compared to the placebo arm (95.0 vs. 117.4 mg/dL, *p*-value = 0.04). TGs were then cleared from the circulation; at 6 h, the serum TG level of the experimental food arm was significantly lower than that of the placebo arm (*p*-value = 0.01) (Figure 3a). Comparison of the 6 h postprandial TG iAUCs adjusted for age, gender, BMI and sequence between the intervention arms is shown in Figure 3b. A significant decrease (from 389.0 to 323.6 mg h/dL, 16.8%, *p*-value = 0.03) was detected in the TG iAUCs of the experimental food arm, comparing to the placebo arm. The effects of age, gender, BMI and sequence were insignificant.

### 3.4. Postprandial RLP Cholesterol Responses

**Figure 4:**
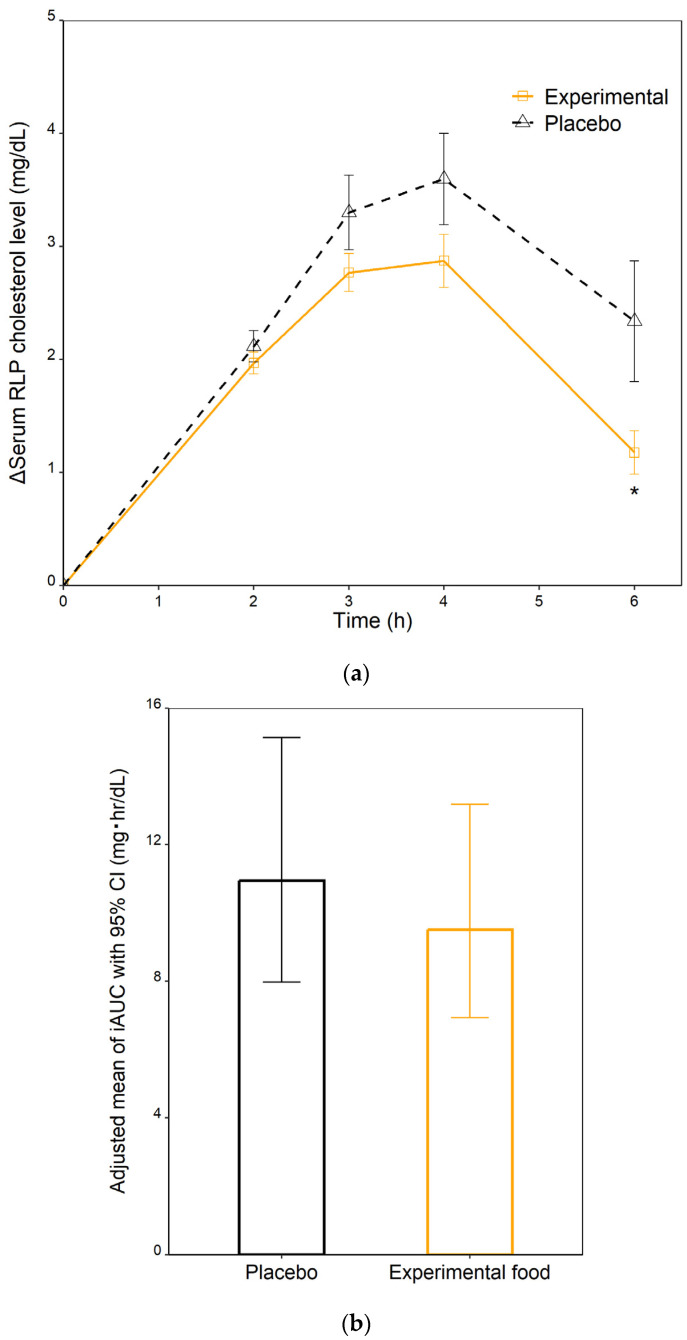
Postprandial remnant-like lipoprotein particle (RLP) cholesterol responses to ingestion of the experimental food or placebo and the standardized high-fat diet. (**a**) Time course of the incremental postprandial serum RLP cholesterol concentrations. Values are mean ± standard error (SE). Statistical analysis by two-tailed, paired *t*-tests. * *p*-value < 0.05. (**b**) Incremental area under the curve (iAUC) of postprandial serum RLP cholesterol. Values are covariate-adjusted mean ± 95% confidence interval (CI) and were compared by using a mixed-effects model with intervention, gender, age, body mass index (BMI) and sequence as fixed variables and subject as a random effect.

The postprandial RLP cholesterol responses, which were measured as the incremental serum levels of RLP cholesterol relative to the baseline/fasting values (denoted by Δ) at sequential time points and the iAUCs of RLP cholesterol, are illustrated in Figure 4. The baseline/fasting serum levels of RLP cholesterol were 3.6 ± 0.3 mg/dL and 4.2 ± 0.5 mg/dL for the experimental food and placebo arm, respectively. In response to a dietary fat intake, the serum level of RLP cholesterol of the experimental food arm increased at a plausibly lower rate than that of the placebo arm. The experimental food treatment contributed to significantly reduced incremental RLP cholesterol level compared to the placebo at 6 h (1.18 vs. 2.34 mg/dL, *p*-value = 0.03) (Figure 4a). Comparison of the RLP cholesterol iAUCs adjusted for age, gender, BMI and sequence between the intervention arms are shown in Figure 4b. Treatment with the experimental food induced a nonsignificant reduction in the RLP cholesterol iAUC as compared to placebo (10.9 vs. 9.5 mg h/dL, *p*-value = 0.14). The effects of age, gender, BMI and sequence were insignificant.

### 3.5. Other Postprandial Lipidemic Parameters

The effects of treatment with the experimental food vs. placebo on the postprandial serum concentrations of other lipidemic parameters and blood glucose are summarized in Table 4. Treatment with the experimental food significantly reduced the serum concentration of phospholipids at 3 h and 6 h following ingestion of the high-fat meal and that of βLPs at all postprandial time points. On the contrary, a significant increase in the serum concentration of HDL cholesterol associated with the experimental food arm was observed at 6 h following the fat intake. The postprandial serum concentrations of FFAs, total cholesterol, LDL cholesterol, and glucose did not differ significantly at any time points between the intervention arms.

### 3.6. Safety Endpoint

The sesame meal extract-containing experimental food was well tolerated; there was no withdrawal attributable to the experimental food after the intervention started. No adverse events, particularly gastrointestinal disorders (e.g., oily, loose stools, and frequent bowel movements), were reported throughout the study.

## 4. Discussion

PL inhibition has been established as a safe and effective strategy to treat obesity. To date, a tremendous number of chemically diverse PL inhibitors of varying degrees of activity have been identified from a wide range of natural sources such as plant extracts and microbial products. Despite maintained interest in the modulation of intestinal lipid handling by targeting PL, very few of the PL inhibitors have found their way into clinical research [5,9]. One of the few is apple polyphenol extract, the PL inhibitory activity of which is attributable to the presence of oligomeric procyanidins. The oral administration of 600 mg of the extract was shown to significantly inhibit the postprandial elevation of TGs in serum in 6 non-hypertriglyceridemic subjects. One obvious limitation of this study was the small sample size, and neither the AUCs nor iAUCs of postprandial TGs were evaluated [10]. *Salvia officinalis* L. leaf extract has been reported to be a PL inhibitor in vitro and effective in lowering lipid absorption in mice. In a trial in patients with hyperlipidemia, *Salvia officinalis* L. leaf extract was found to reduce the values of fasting TGs and several other lipidemic parameters; however, since neither the postprandial lipemic, particularly TG, responses, nor fecal fat excretion were monitored, whether the antihyperlipidemic action of the extract was attributable to its PL inhibitory activity remains unclear [11]. Tea polyphenols have been shown to reduce postprandial hypertriglyceridemia in non-hypertriglyceridemic and mildly hypertriglyceridemic individuals through PL inhibition [12,13]. In addition, 3-month treatment with a green tea extract was reported to be effective in reducing body weight and waist circumference in moderately obese patients, but the parameters related to lipid metabolism were not measured [14]. Taken together, it is evident that there is a scarcity of clinical studies of natural product-derived PL inhibitors. We had previously reported that the ethanolic extract of sesame meal was a potent PL inhibitor in vitro with its activity hinging exclusively on two non-esterified FFAs—LA and OA, and that the administration of a mixture of these FFAs markedly reduced the elevation of serum TGs after a high oral fat load in rats [8]. To investigate if the outcome obtained in rodent models could be translated to humans, in the present study, we further assessed the effects of the sesame meal extract as a carrier of free LA and OA on postprandial lipemic responses in human subjects. The results of the study demonstrated that a single oral administration of 90 mg of the free LA and OA-enriched sesame meal extract was effective in reducing the absorption of dietary fat after ingestion of a standardized high-fat meal, as measured by attenuated postprandial TG excursions, in non-hypertriglyceridemic, nonobese individuals. Treatment with the extract was also shown to impede the postprandial rise of serum RLP cholesterol, phospholipids, and βLPs, and to enhance the HDL cholesterol response. The present study is appropriately powered and the first trial that demonstrated the efficacy of free LA and OA-enriched natural products in modulating dietary fat absorption and the consequent postprandial lipemic responses by inhibiting PL in healthy individuals. Furthermore, the free LA and OA-enriched sesame meal extract was effective at a lower dose (90 mg per meal) compared to those of the aforementioned extracts or compounds (>200 mg per meal for solid or 500 mL per meal for liquid), indicating the possibly greater PL-inhibitory potency of the sesame meal extract conferred by free LA and OA, although explicit comparisons were difficult.

Humans spend most of diurnal time in a postprandial state, and it is increasingly acknowledged that disease development is essentially associated with the dysregulation of substrate fluxes in the immediate postprandial period [15]. Epidemiological studies have revealed that elevated postprandial TG levels pose increased risks for a myriad of chronic conditions, particularly atherosclerotic cardiovascular diseases [2,16,17,18,19], and the associated mortality [19,20,21] in men and women. The postprandial period, especially after a fatty meal, is characterized by a rise in circulating TGs, which results in an increase in TG-rich chylomicrons and TG-rich very-low-density lipoprotein (VLDL), which are collectively known as TG-rich lipoproteins (TRLs) [22,23]. It is established that the effects of elevated postprandial TGs on the pathophysiology of atherosclerotic cardiovascular diseases are substantially mediated by RLPs, the lipolytic products derived from TRLs, rather than TGs or TRL themselves [17,18,19,20,21]. RLPs, carrying 5 to 20 times more cholesterol per particle than LDL particles, can penetrate the endothelial barrier and be readily taken up in an unregulated fashion by scavenger receptors expressed by resident macrophages in the subendothelial space, facilitating cholesterol deposition and the development of atherosclerosis [24]. Herein, we reported that oral intake of the free LA and OA-enriched sesame meal extract led to reduced mean incremental RLP cholesterol level at all postprandial time points and a statistically significant difference was observed at 6 h, although statistical significance was not achieved for the iAUC of RLP cholesterol. Meanwhile, intake of the extract also contributed to significant decreases, at all postprandial time points, in the concentration of circulating βLP or LDL particles, which are more atherogenic than the LDL particles in the fasting state [25] and regarded as a stronger predictor of the incidence of cardiovascular events than either LDL cholesterol or non-HDL cholesterol [26]. As both RLPs and LDL are derived from TRLs, the suppressed postprandial rise in RLP cholesterol and LDL particles observed after intake of the extract points to a postprandial state of attenuated TRL responses due to ameliorated triglyceridemia. Moreover, we observed a significantly higher HDL cholesterol level at 6 h after consumption of the high-fat meal along with the free LA and OA-enriched extract. It is known that elevated postprandial TGs and TRLs favor the exchange of cholesteryl esters and TGs between HDL and TRLs, resulting in TG enrichment of HDL, reducing HDL cholesterol and exacerbating HDL dysfunction [18,23,27,28,29]. Therefore, we speculate that the higher postprandial HDL cholesterol level associated with the extract intake is likely a result of the attenuated postprandial triglyceridemia and TRL elevation and the thereby reduced transfer of cholesteryl esters from HDL to TRL. In case of high TG availability, similar to HDL, LDL particles are also modified by TG transfer, resulting in the formation of small dense LDL and a slight decrease in the cholesterol components of LDL [29], which is consistent with our observation in this study. However, it seemed that a single dietary intervention with the sesame meal extract did not alter the trajectory of postprandial LDL cholesterol in a significant manner comparing to the placebo. In addition, the postprandial rise in phospholipids was observed for both arms as a result of the digestion and absorption of phospholipids present in the standardized high-fat meal. Lower levels of postprandial phospholipids were observed in association with the intake of the extract. The cause of this alteration remains to be further clarified as postprandial phospholipid responses relate to various factors, for instance, the phospholipase-mediated digestion of dietary phospholipids and the transport and subsequent remodeling of the digestive products of phospholipids [30,31]. Finally, it should be noted that despite the promising results about the attenuating effects of dietary intervention with the free LA and OA-enriched sesame meal extract on postprandial lipemia, these results do not permit us to draw conclusions regarding its effects on the lipoprotein subclass pattern in real-life scenarios, wherein the meals are sequential.

## 5. Conclusions

In conclusion, our postprandial study in healthy participants revealed that a single oral dose of the free LA and OA-enriched sesame meal extract significantly reduced postprandial triglyceridemia after a high fat load. This reduction not only curbs excess energy intake, which is a main driver of obesity, but may also contribute to the attenuation of the postprandial increase in atherogenic lipoproteins. Since we have verified in vitro that the activity of the sesame meal extract against PL can be exclusively attributed to its constituent free LA and OA, the results of the present study suggest that free LA and OA and the natural products rich in these FFAs may represent a class of promising nutraceuticals that may serve as molecular modulators of intestinal lipid handling. Despite the great number of natural product-derived PL inhibitors reported in the literature, they have been scarcely studied in humans. The present study is the first trial that has demonstrated the clinical efficacy of free LA and OA-rich natural products in modulating postprandial lipemic responses plausibly via PL inhibition in healthy individuals, adding to the sum of clinical evidence of using natural product-derived PL inhibitors to tackle obesity and related disorders. Moreover, the findings of this study are particularly important in that free LA and OA are widely present in various natural products so that these compounds can be readily accessed and utilized, providing a feasible approach for routine postprandial lipid handling. Further investigations are hence warranted to explore the mid-to-long term efficacy and safety profile of the sesame meal extract and other free LA and/or OA-rich nutraceuticals in the prevention or treatment of obesity and related conditions for the general population.

## Figures and Tables

**Table 1:** Nutritional composition of the experimental food and the placebo.

	Nutritional Value per Serving	
	Experimental Food	Placebo
Carbohydrate (g)	0.791	0.849
Protein (g)	0.043	<0.001
Fat (g)	0.008	<0.001
Dietary fiber (g)	<0.005	<0.005
Water (g)	0.053	0.051
Sodium (mg)	0.032	0.067
Ash * (g)	0.005	<0.001
Free linoleic acid (LA) (mg)	2.100	–
Free oleic acid (OA) (mg)	1.900	–
Energy (kcal)	3.411	3.393

* Ash refers to the inorganics remaining after complete removal of moisture, volatiles and organic matter by heating.

**Table 2:** Description of the standardized high-fat meal.

Item	Manufacturer	Quantity	Nutritional Value per Unit of Item			
			Fat (g)	Protein (g)	Carbohydrate (g)	Energy (kcal)
Tasmanian beef Japanese-style hamburger	Aeon Topvalu, Japan	1	17.0	20.6	14.8	288
Neo Butter Roll	Fujipan, Japan	2	9.3	2.8	17.0	163
Hashed potatoes	Heinz, Japan	1	8.2	1.4	14.0	136
Total			43.8	27.6	62.8	750

**Table 3:** Baseline characteristics of all randomized and exposed participants.

Characteristics	All Randomized and Exposed (*n* = 30)	All Analyzed (*n* = 26)
Gender, *n* (%)		
Male	18 (60%)	14 (54%)
Female	12 (40%)	12 (46%)
Age (years)	35.7 ± 8.3 (min 24, max 55)	36.0 ± 8.1 (min 24, max 55)
Body Mass Index (BMI, kg/m^2^)	23.1 ± 2.3	22.8 ± 2.2
Fasting lipidemic parameters		
TG (mg/dL)	91.8 ± 44.6	81.8 ± 37.9
RLP-C (mg/dL)	4.5 ± 2.5	3.9 ± 2.1
FFAs (μEq/L)	409.7 ± 143.8	338.7 ± 128.0
Total cholesterol (mg/dL)	185.1 ± 27.0	182.2 ± 27.5
Phospholipids (mg/dL)	189.9 ± 19.1	187.0 ± 18.5
βLPs (mg/dL)	311.2 ± 83.6	293.9 ± 75.4
HDL cholesterol (mg/dL)	56.9 ± 9.3	57.8 ± 9.25
LDL cholesterol (mg/dL)	111.0 ± 25.6	108.3 ± 26.5
Fasting blood glucose (mg/dL)	90.2 ± 7.0	89.6 ± 5.7

Values are mean ± standard deviation (SD). Abbreviations: TG, triglyceride; FFAs, free fatty acids; βLPs, beta lipoproteins; HDL, high-density lipoprotein; LDL, low-density lipoprotein.

**Table 4:** Postprandial serum concentrations of other lipidemic and glycemic parameters.

Parameter	Intervention	Serum Concentration				
		0 h	2 h	3 h	4 h	6 h
FFAs (μEq/L)	Placebo	362.9 ± 24.4	257.1 ± 20.2	404.7 ± 36.3	458.4 ± 37.0	646.5 ± 42.4
	Experimental food	388.2 ± 22.4	245.0 ± 16.1	379.8 ± 24.9	451.3 ± 27.4	654.4 ± 28.3
Total cholesterol (mg/dL)	Placebo	182.2 ± 5.0	176.8 ± 4.6	181.9 ± 4.8	182.8 ± 4.9	183.5 ± 4.8
	Experimental food	181.4 ± 5.4	176.5 ± 5.1	179.5 ± 4.7	180.9 ± 4.8	182.5 ± 4.8
Phospholipids (mg/dL)	Placebo	189.7 ± 3.9	193.1 ± 3.8	201.0 ± 4.2	204.4 ± 4.7	209.9 ± 5.0
	Experimental food	187.5 ± 3.6	190.3 ± 3.8	196.2 ± 3.6 * (*p*-value = 0.03)	201.8 ± 3.6	204.2 ± 4.0 * (*p*-value = 0.02)
βLPs (mg/dL)	Placebo	303.9 ± 16.3	379.3 ± 19.8	400.2 ± 25.2	382.7 ± 26.8	340.5 ± 22.8
	Experimental food	291.5 ± 13.8	359.8 ± 17.3 * (*p*-value = 0.01)	367.8 ± 19.8 * (*p*-value = 0.01)	356.9 ± 20.5 * (*p*-value = 0.03)	313.3 ± 17.4 * (*p*-value = 0.003)
HDL cholesterol (mg/dL)	Placebo	57.4 ± 1.7	52.6 ± 1.7	53.5 ± 1.8	54.2 ± 1.9	56.2 ± 1.8
	Experimental food	58.2 ± 1.6	53.8 ± 1.6	54.7 ± 1.6	55.4 ± 1.5	57.9 ± 1.7 * (*p*-value = 0.03)
LDL cholesterol (mg/dL)	Placebo	107.4 ± 5.0	101.5 ± 4.5	100.8 ± 4.3	102.0 ± 4.3	105.3 ± 4.5
	Experimental food	107.8 ± 5.1	101.8 ± 4.6	101.2 ± 4.2	102.6 ± 4.4	106.7 ± 4.6
Glucose (mg/dL)	Placebo	90.4 ± 1.2	86.3 ± 2.3	88.1 ± 1.8	89.3 ± 1.3	87.2 ± 1.0
	Experimental food	89.1 ± 1.1	84.3 ± 2.7	88.5 ± 1.8	88.4 ± 1.2	87.3 ± 0.9

Values are mean ± standard error (SE). Statistical analysis by two-tailed, paired *t*-tests. * *p*-value < 0.05. Abbreviations: FFAs, free fatty acids; βLPs, beta lipoproteins; HDL, high-density lipoprotein; LDL, low-density lipoprotein.

## Data Availability

The dataset generated during and analyzed during the present study is not publicly available. De-identified participant data are however available from the corresponding author upon reasonable request.
